# Analysis of *RP2* and *RPGR* Mutations in Five X-Linked Chinese Families with Retinitis Pigmentosa

**DOI:** 10.1038/srep44465

**Published:** 2017-03-15

**Authors:** Jingjing Jiang, Xiaofei Wu, Di Shen, Lijin Dong, Xiaodong Jiao, J. Fielding Hejtmancik, Ningdong Li

**Affiliations:** 1 MOE Key Laboratory of Major Diseases in Children, Beijing Children’s Hospital, The Capital University of Medical Science, Beijing, China; 2 Tianjin Medical University, Tianjin, China; 3 Xi’An Ophthalmological Hospital, Xi’An No 1 Hospital, Xi’An, 710002 Shanxi Province, China; 4 Genetic Engineering Core, NEI, NIH, Bethesda, MD, USA; 5 OGVFB, NEI, NIH, Bethesda, MD, USA

## Abstract

Mutations in RP2 and RPGR genes are responsible for the X-linked retinitis pigmentosa (XLRP). In this study, we analyzed the RP2 and RPGR gene mutations in five Han Chinese families with XLRP. An approximately 17Kb large deletion including the exon 4 and exon 5 of RP2 gene was found in an XLRP family. In addition, four frameshift mutations including three novel mutations of c.1059 + 1 G > T, c.2002dupC and c.2236_2237del CT, as well as a previously reported mutation of c.2899delG were detected in the RPGR gene in the other four families. Our study further expands the mutation spectrum of RP2 and RPGR, and will be helpful for further study molecular pathogenesis of XLRP.

Retinitis pigmentosa (RP) is a clinically and genetically heterogeneous group of retinal dystrophies characterized by photoreceptor cell degeneration. The prevalence of RP is about 1/4000 ~1/5000 according to studies in different populations^1^. The clinical features comprise night blindness, progressive constriction of visual field with age, and fundus changes including attenuation of the retinal arterioles, bone spicule-like pigment deposits in the mid-peripheral retinal and waxy pallor of the optic disc. Affected individuals often have severely abnormal or non-detectable rod responses in the electroretinograms (ERG) recordings even in the early stage of the disease^2^.

RP is inherited as an autosomal dominant trait in about 15–20% of families, an autosomal recessive trait in about 20–25% of families, and an X-linked trait in about 10–15% of families, with digenic patterns occurring rarely. In addition, about 50% of RP cases are sporadic, although many of these cases may represent autosomal recessive RP^1^. X-linked RP (XLRP) is the most severe form of RP in terms of age of onset and progression, with affected males generally showing more severe clinical features than affected females. They usually experience night blindness and loss of dark adaptation in the first decade of life. Peripheral visual fields begin to constrict in the second decade and complete loss of central visual acuity generally occurs in the fourth or fifth decade. In contrast, female carriers show variable clinical symptoms of the disease with visual impairment usually beginning in middle age^3^,^4^.

*RPGR* (OMIM #312610), *RP2* (OMIM #312600), and *OFD1* (OMIM #300170) are three genes in which mutations can cause XLRP. Additional genetic loci for XLRP, including RP6 on Xp21.3^5^, RP24 on Xq26. and RP34 on Xq28^6^ have been mapped, but the respective genes have not yet been identified. It was suggested that approximately 70–90% of XLRP cases are caused by RPGR mutations and 6–20% of XLRP are caused by RP2 mutations^3^,^7^,^8^,^9^,^10^,^11^,^12^,^13^,^14^,^15^. Here we report molecular genetic analysis of five Chinese families with X-linked RP showing one large deletion in *RP2* and four frameshift mutations in *RPGR* were detected in these five families.

## Materials and Methods

### Patient Ascertainment

Five Han Chinese families (XLRP001,XLRP002, XLRP003, XLRP004 and XLRP005) with X-linked RP were recruited through the Ophthalmic Genetics Clinic of Beijing Children Hospital, Beijing, China. IRB approval was obtained for this study from the CNS IRB of the National Institutes of Health, Bethesda, MD, USA and Beijing Children Hospital, Beijing, China. The participating subjects gave informed written consent, consistent with the tenets of the Declaration of Helsinki. The five families described in this study are from Beijing and Tianjin, China. General medical, genetic, and ophthalmological histories were obtained by interviewing family members. All family members underwent a complete ophthalmologic examination including best corrected visual acuities, and examination of the anterior segment, vitreous and fundus. Full field ERGs were recorded according to the standards of the International Society for Clinical Electrophysiology of Vision (http://www.iscev.org). Blood samples were collected from affected and unaffected family members. DNA was extracted as described by Smith *et al*.^16^.

### Genotyping and Linkage Analysis

Linkage analysis was performed on two families (XLRP001 and XLRP002) with a sufficient number of affected individuals. Initially, an excluded linkage screen was performed for the possible adRP loci with 56 highly polymorphic fluorescent markers flanking each of 28 adRP loci, including D1S2726-PRPF3-D1S498, D1S484-SEMA4A-D1S2878, D1S2890-RPE65-D1S230, D1S2842-OR2W3-D1S2836, D2S286-ASCC3L1-D2S160, D2S206-SPP2-D2S338, D3S1267-RHO-D3S1292, D6S422-RDS-D6S1610, D6S1610-PRPH2-D6S257, D6S262-RP63-D6S1565,D6S1650-GUCA1B-D6S257, D7S516 -PAP1 (RP9)-D7S484, D7S493-KLHL7-D7S516, D7S486-IMPDH1-D7S530, D8S258-RP1-D8S285,D9S171-TOPORS (RP31)-D9S161, D9S1677-PRPF4-D9S1776, D10S537-HK1-D10S1686, D11S4191-BEST1-D11S987,D11S4197-ROM1-D11S987, D14S283-NRL-D14S275, D14S68-RDH12-D14S74, D15S153-NR2E3-D15S131, D17S831-PRPF8-D17S938, D17S787-CA4-D17S944, D17S785-FSCN2-D17S784, D19S420-CRX-D19S902, D19S572-PRPF31-D19S418, D20S171-PRPF6-D20S173. After these adRP loci were excluded, an X chromosome linkage scan was performed on these two families using markers from the ABI MD10 panel (Applied Biosystems, Foster City, CA). Multiplex polymerase chain reactions (PCR) were carried out as previously described^17^. PCR products from each DNA sample were pooled and mixed with a loading cocktail containing HD-400 size standards (PE Applied Biosystems, Foster City, CA) and loading dye. The resulting PCR products were separated on an ABI3130 sequencer and analyzed using GENEMAPPER 4.0 (PE Applied Biosystems, Foster city, CA).

Two point linkage analyses were performed using the FASTLINK version of the LINKAGE Program Package via the easyLINKAGE program^18^,^19^,^20^.The marker order and distances between the markers were obtained from the Généthon database (http://www.genethon.fr/). Haplotypes were constructed manually using the Cyrillic program to define the borders of the cosegregating region.

### DNA sequencing

Sequence analysis to two candidate genes of *RP2* and *RPGR* were carried out by direct DNA sequencing. Primers were designed from exon and intron sequences for amplification of whole coding regions and exon-intron boundaries of *RP2* and *RPGR.* Exon 2 of *RP2* and exon ORF15 of *RPGR* were split into two and four overlapping amplicons respectively. All PCR primer sequences are listed in the Table 1. Amplifications were carried in 25 μL of standard PCR buffer containing 1.5 mM MgCl_2_, 0.2 mM of each dNTP, 0.5 μM of each primer, 1 U of Taq polymerase, and 50 ng of DNA. The amplification program consisted of an initial 2 min denaturation at 98 °C followed by 30 cycles of 30 s at 94 °C, 30 s at 55 °C, 1 min at 72 °C, and a final 7 min extension step at 72 °C.

### Long-distance inverse PCR

**Figure 1:**
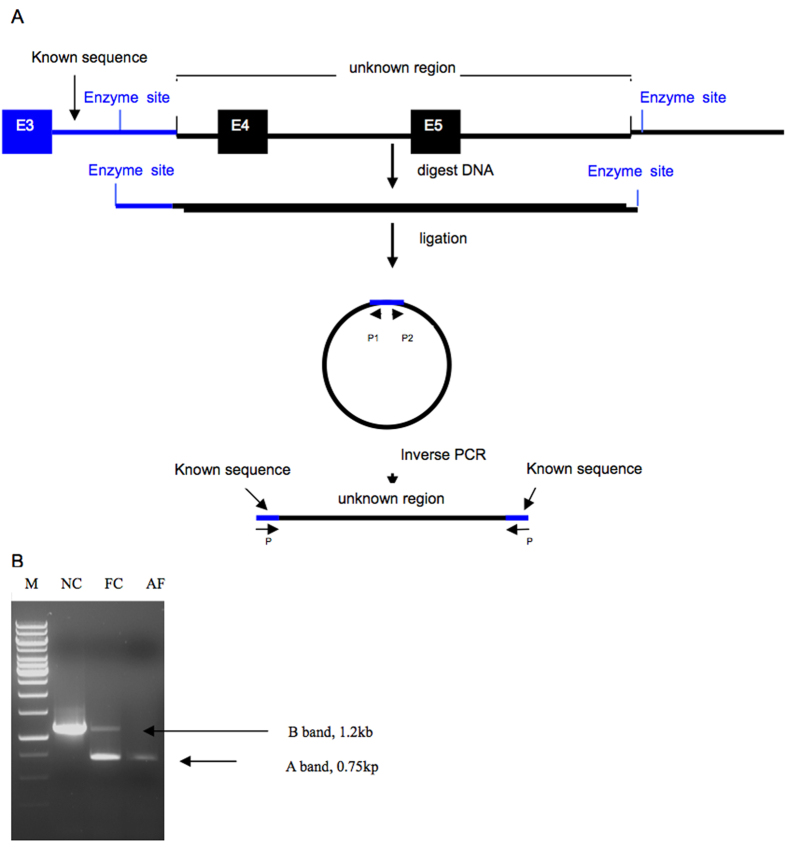
(**A**) Schematic diagram of the self-ligation of restriction endonuclease-digested DNA fragments with long-distance inverse PCR. The blue line denoted the known sequence in intron3 of RP2 gene. The unknown region is a particular region to be investigated containing the breakpoints of the deletion. Capital E represents the exon. Small arrows with the letter P represent the sites of the primer. (**B**) After digestion with BglII and then circularization with T4 DNA ligase followed by inverse PCR, an approximately 750 bp fragment (A band) was produced from an affected male (AF) and a female carrier (FC), while an approximately 1.2 kb fragment (B band) was produced from the female carrier and a normal control (NC).

After failure of amplification of exons 4 and 5 of RP2 in all affected males in the XLRP001 family, a long-distance inverse PCR was designed to identify the boundaries of the deleted region in *RP2*^21^,^22^. The primers for inverse PCR were selected from the region inside the restriction enzyme site of known DNA sequence adjacent to the end of the unknown DNA sequences and oriented in the reverse direction (Fig. 1A), and the sequences of the primers were used as followed: P1(sense), 5′-TGAAGAGTCTTCAGACTCTTCCCATGATTTTTATT-3′; P2 (antisense), AGCCCTTTCAGTGGTTCTTGATGTAAAATTAA-3′. Five microgram aliquots of genomic DNA from each sample were digested with 5 different restriction endonucleases, including AvRII, BglII, EcorI, SmaI, and StuI, precipitated with ethanol, and dissolved in 10 μl H_2_O. The digested DNA was circularized by self-ligation in 20 μl of reaction mixture containing 2 μL of ligation buffer and 2 μL of T4 DNA ligase (Promega, Madison, WI) at 4 °C for 16 hours. One microliter of the self-ligated DNA was used as a template for inverse PCR (IPCR).

**Figure 2:**
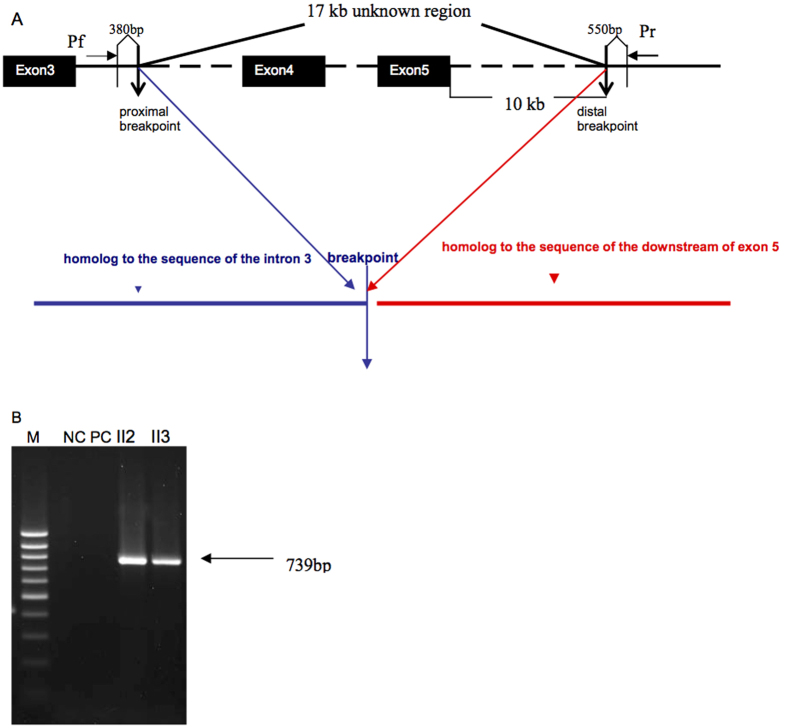
Long range PCR and sequence analysis. (**A**) Schematic diagram of the 16.529 kb deletion of the RP2 gene and sequence analysis of the 739 bp band from the long-range PCR. The broken line represents the region of deletion. The site of the primer Pf was located 380 bp upstream of the proximal breakpoint, while the primer Pr was located 550 bp downstream of the distal breakpoint. (**B**) Agarose gel electrophoresis of PCR products long-range PCR. A 739 bp band was produced from an affected male (II2, XLRP001) and a female carrier (II3, XLRP001). NC means negative control. PC denotes a positive control.

Subsequently, to confirm the results of the inverse PCR, long range PCR amplification was performed using 5U of LA Taq polymerase (Takara, Japan) in a 50 μL volume containing 2.5 mM MgCl_2_, 0.4 mM of each dNTP, 0.5 μM of each primer, and 50 ng of DNA. The primers used for long range PCR were as follows: Pf (sense), 5′-TGAAGAGTCTTCAGACTCTTCCCATGATTTTTATT-3′; Pr (antisense), AGCCCTTTCAGTGGTTCTTGATGTAAAATTAA-3′. Primer Pf was located 380 bp upstream of the proximal breakpoint in intron3, while the primer Pr was located 550 bp downstream of the distal breakpoint, approximately 10 kb beyond exon5 (Fig. 2A). In unaffected individuals, the resulting PCR product is predicted to be about 17.35 kb, while in individuals with the deletion the amplicons should be 930 bp. Conditions for the long-range PCR amplification were an initial 2 min denaturation at 98 °C, followed by 35 cycles of 15 s at 98 °C, 20 min at 66 °C, and a final 10 min extension step at 72 °C.

All PCR products were separated by electrophoresis on 1.2% agarose gels and extracted using the QIAquick Gel Extraction Kit (Qiagen, Valencia, CA). DNA sequencing analysis was performed using the BigDye Terminator Cycle Sequencing V3.1 kit on an ABI PRISM 3130 Genetic Analyzer (Applied Biosystems). Sequencing results were assembled and analyzed using the Seqman program of DNASTAR software (DNASTAR Inc., Madison, WI). Splicing mutations were analyzed by the *Human Splicing Finder (HSF)* program. (http://www.umd.be/HSF/).

## Results

### Clinical Assessment

**Figure 3:**
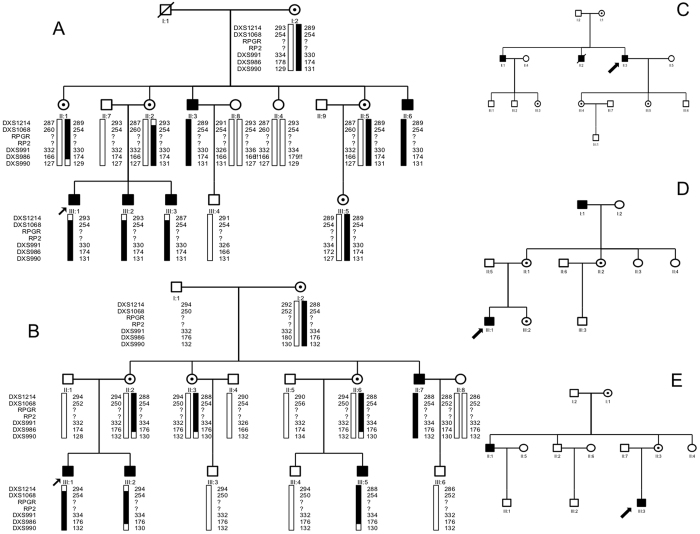
Five Families with X-linked RP. No male-to-male transmission in these families. Squares are males, circles are females, filled symbols are affected individuals,dotted circles are female carriers, a diagonal line through a symbol indicates a deceased family member, and the arrow indicates the proband. The haplotypes with 5 microsatellite markers from the RP2 and RPGR region of chromosome Xp11.3 are shown with alleles forming the risk haplotype are shaded black and alleles not co-segregating with RP are shown in white. (**A**) XLRP001 family; (**B**). XLRP002 family; C. XLRP003 family; (**D**) XLRP004 family; (**E**) XLRP005 family).

**Figure 4:**
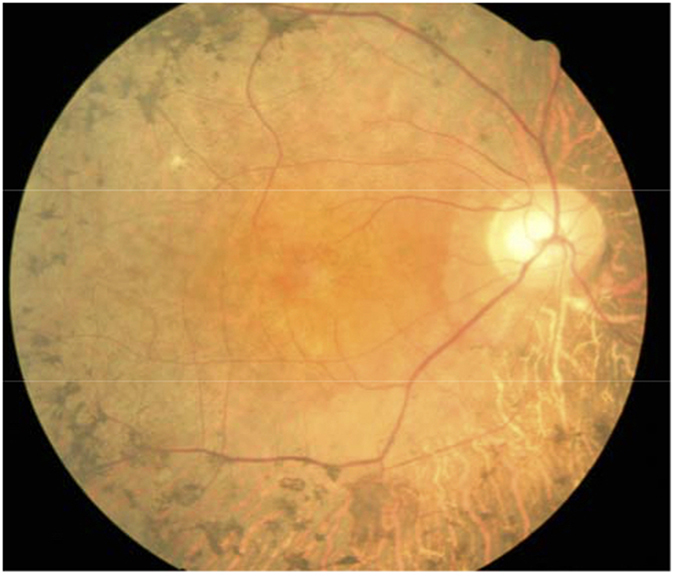
Fundus photograph of the RP patient. Fundus photograph of the right eye from the proband in the family XLRP001, showing typical changes of RP including waxy yellow appearance of the optic disc, attenuation of retinal arteries and bone-spicule pigment deposits in the mid periphery of the retina.

**Figure 5:**
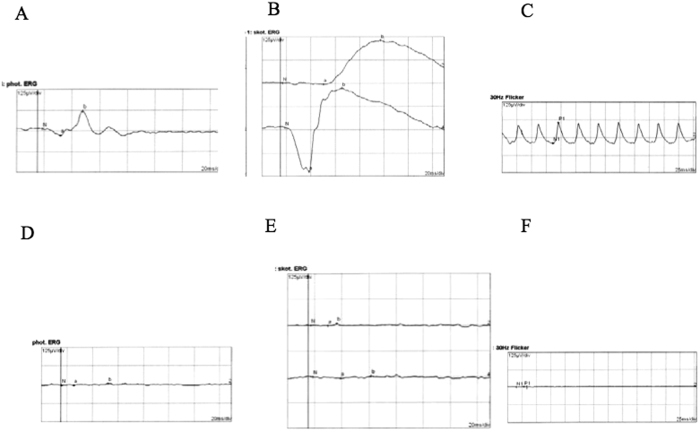
ERG recordings from an affected RP individual and a normal control. Compared with the ERG recordings from a normal individual, an extinguished ERG was recorded from the left eye of the affected individual (the proband in the family XLRP001) under the photopic, scotopic and 30 Hz flicker conditions. (**A** and **D**), photopic ERG from a normal control (**A**) and the patient (**D**); (**B** and **E**), rod (upper) and max (lower) responses under scotopic conditions from a normal (**B**) and an affected individual; C and F, 30 Hz flicker conditions from a normal control (**C**) and the patient (**F**).

There was no male-to-male transmission in these five families (Fig. 3). The disease was transmitted either from a female carrier to an affected son or to an affected or carrier female. The affected males had onset of night blindness around 7–8 years of age, and had fundus changes typical of RP including a waxy, pale optic disc, attenuation of retinal arteries and bone-spicule pigment deposits in the mid periphery of the retina as shown in individual 5, a 25-year-old proband in the family XLRP001 (Fig. 4). Results of his ERG examination showed extinguished activity under either photopic or scotopic conditions (Fig. 5). The clinical evaluations performed for all affected individuals as well as the obligated female carriers are listed in the supplement Table 1.

Initially, 28 genes known to cause adRP when mutated were excluded as causing disease in the families XLRP001 and XLRP002 because microsatellite markers closely flanking each showed lod scores less than −2. Then an X chromosome-wide linkage scan yielded significant lod scores of 2.7 at DXS991 (θ = 0) and 2.4 at DXS986 (θ = 0) for the family XLRP001. A significant lod score of 2.4 was obtained at DXS1068 (θ = 0) for the family XLRP002. Two-point linkage analyses showed that the disease-causing gene of the XLRP001 and XLRP002 families were linked to microsatellite markers in chromosome Xp11.3, where *RP2* and *RPGR* are located. (Fig. 3)

**Figure 6:**
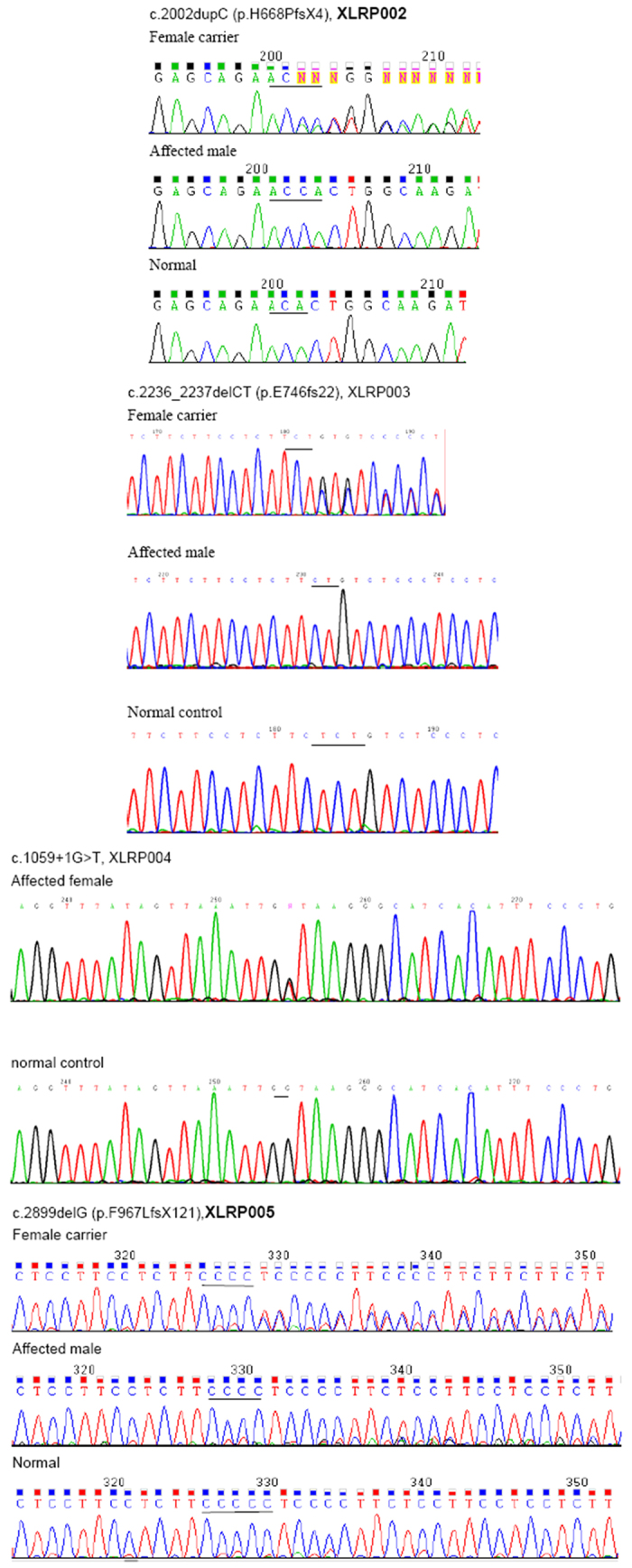
Sequence analysis of RPGR gene for four families with X-linked retinitis pigmentos. Sequencing chromatograms from a female carrier, an affected male and a normal individual in each family. Mutations in RPGR were identified in each of four families: a novel insertion mutation of c.2002dupC (p.H668PfsX4) in the family XLRP002, a novel small deletion of c.2236_2237delCT (p.E746fs22) in the family XLRP003, a novel splicing mutation of c.1059 + 1 G > T in the family XLRP004, and a previously reported mutation of c.2899delG (p.F967LfsX121) in the family XLRP005.

Sequence analysis of the *RP2* and *RPGR* genes showed four frameshift mutations in *RPGR* (Genebank accession number AF286472) in each of four families, including a novel insertion mutation, c.2002dupC (p.H668PfsX4), in family XLRP002, a novel small deletion, c.2236_2237delCT (p.E746fs22), in family XLRP003, a novel splicing mutation, c.1059 + 1 G > T, in family XLRP004, and a previously reported deletion, c.2899delG (p.F967LfsX121), in family XLRP005^23^ (Fig. 6).

**Figure 7:**
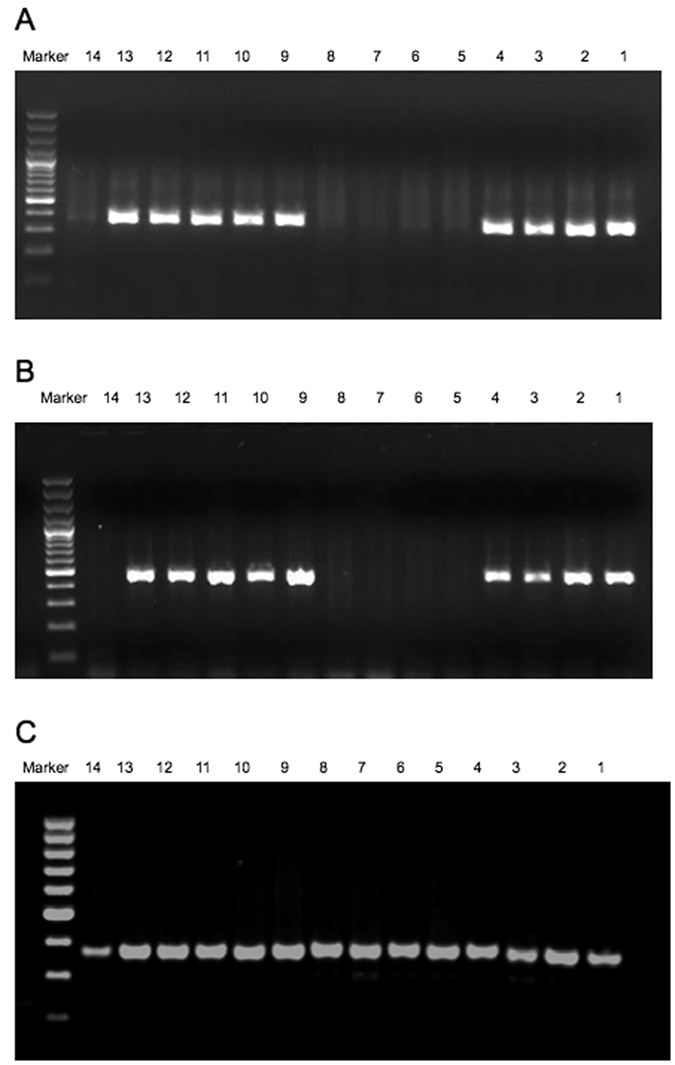
Agarose gel electrophoresis of PCR products of exon4 (**A**), and the coding region of exon5 (**B**). All affected males in the family XLRP001 (individuals 5,6,7,8, and14) show absence of amplification due to hemizygous deletion of this region, but show presence of amplification of exon3 (**C**).

However, no mutations were identified in *RPGR* or exons1 to 3 of *RP2* in affected individuals or female carriers in the family XLRP001. Repeated failure of PCR amplification for exon 4 and the coding region of exon 5 of *RP2* was observed in all affected males as shown in Fig. 7(A and B), suggesting the possibility of a deletion encompassing this region of *RP2* in affected individuals.

In order to identify the proximal and distal deletion breakpoints of the suspected deletion, sets of primers located approximately every 1 kb between exons 3 and 4 were used for PCR amplification of genomic DNA from an affected male. The results indicated that the proximal deletion breakpoint was within about 3,562 bp upstream from exon4 of the RP2 gene (data not shown). Then self-ligation of restriction endonuclease-digested DNA fragments coupled with long-distance inverse PCR was used to localize both the proximal and distal deletion breakpoints. Genomic DNA was digested with BglII followed by circularization of the restriction fragments using T4 DNA ligase and inverse PCR (Fig. 1A). An approximately 750 bp amplicon was produced from both an affected male and a female carrier, while an approximately 1.2 kb amplicon was produced from the female carrier and a normal control (Fig. 1B). The bands were excised from the gel and purified separately and were then sequenced directly, and compared with the *RP2* gene map. The 1.2 kb fragment aligned with the complete sequence inside the intron3 of RP2, while DNA in the 750 bp band aligned with two distinct regions of *RP2* DNA. Approximately 350 bp matched DNA sequences located within intron3, while the remaining 400 bp matched genomic DNA sequences located 11–12 kb downstream from exon5, identifying the distal breakpoint. Long range PCR amplification with primers Pf and Pr was next used to identify the precise location of the proximal and distal breakpoints (Fig. 2A). The PCR product in control individuals was predicted to be approximately 17 kb, based on the locations of the primer Pf and Pr in the RP2 gene map. Long range PCR amplification from the affected male and the female carrier produce a 739 bp fragment (Fig. 2B), but no PCR product was amplified from a normal control probably because it was too large to be amplified in the presence of GC-rich sequence. Sequencing of the 739 bp indicates a 16,618 bp deletion in all affected males, extending from 3,562 bp proximal of exon 4 and extending to 10790 bp distal to exon 5, thus encompassing all of exons 4 and 5 (Fig. 2A). None of the above three novel mutations in *RP2* and *RPGR* were seen in 50 ethnically matched control individuals (100 chromosomes).

## Discussion

*RP2* (MIM 312600) consists of 5 exons spanning approximately 45 kb on chromosome Xp11.3–11.23 and encodes a ubiquitously expressed protein of 350 amino acids^24^. In the cell, the RP2 protein is localized at the plasma membrane via both myristoylation and palmitoylation at its N-terminus^24^,^25^. The function of the RP2 protein is not completely clear. It is suggested that RP2 can stimulate the the GTPase activity of tubulin in the presence of cofactor D^24^. RP2 also interacts with ADP-ribosylation factor-like-3 (Arl3) in a nucleotide- and myristoylation-dependent manner^26^. RP2 is a GTPase activating protein for the small GTPase Arl3, and they work together to link the cell membrane with cilia and the cytoskeleton in photoreceptors as part of the cell signaling or vesicular transport machinery. In addition, the C-terminus of RP2 has sequence similarity to nucleoside diphosphate kinases (NDK)^27^,^28^. NDKs are involved in the transfer of phosphate groups from ATP to nucleoside and deoxynucleoside diphosphates, and its isozymes may play an important role in cellular growth and development. It also has been suggested that RP2 contains an intrinsic 3′ to 5′ exonuclease activity and might potentially function in DNA repair processes^29^.

**Figure 8:**
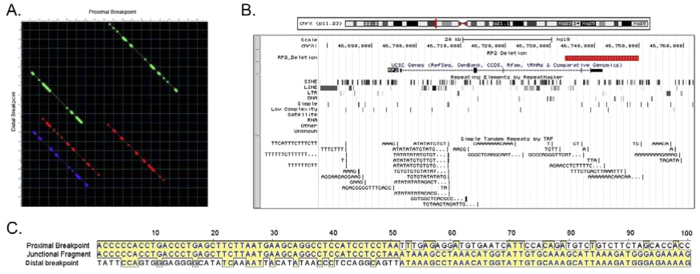
Repetitive sequences and homology between the proximal and distal breakpoints in the RP2 gene. (**A**) Dot blot of 100 bp surrounding the proximal and distal breakpoints with run of greater than 50% homology shown, color coded with red highest and purple lowest. (**B**) UCSC browser view of the RP2 gene region showing the deleted region in red, the SINE (including Alu), LINE, and other repetitive sequences, and simple tandem repeats. (**C**) alignment of the 100 bp surrounding the proximal and distal breakpoints with no gaps. The breakpoint is seen at position 51, with the junctional fragment showing homology to the proximal region before and the distal fragment after that point, but minimal homology among all 3 sequences, and no short regions of homology at the breakpoint itself, the nearest being the GA dinucleotide at position 56–57.

Currently, more than 20 different mutations have been identified in *RP2*, including missense mutations, nonsense mutations, frameshifts, insertions, and deletions^7^. These mutations have been described in exons 1, 2, 3 and 4, but to date no mutation has been detected in exon 5, although a 12.5 Kb deletion including exon 4 has been seen in an isolated male with RP^30^. Mutations in the N-terminus of RP2 can abolish localization to the plasma membrane, whereas C-terminal protein truncation mutations can lead to scattered fluorescent foci in the cytoplasm^24^,^25^,^26^. Loss of the RP2 protein and/or aberrant intracellular distribution appear to be the basis for the photoreceptor cell degeneration in most RP2 cases^24^. In this study, the 16,529 bp deletion identified in this family results in deletion of exons 4 and 5. A truncated mutant protein with 294 amino acids is predicted to result from the gene lacking exon4 and transcription region of exon5. While the mutant protein is predicted to escape nonsense mediated decay, this deletion results in the loss of most part of the C- terminal domain of RP2 protein, which might lead to misfolding and subsequent degradation of the non-functional protein in the photoreceptor cell^24^,^31^. The genomic region around RP2 is rich in simple repeats, LINE, and SINE sequences, including ALUs (Fig. 8B), which tend to increase rearrangements^32^. However, there are no large regions of homology immediately around the recombination site (Fig. 8A) as is seen in nonallelic homologous recombination. This suggests the recombination process might be nonhomologous end joining, although are there no short regions of homology at the recombination site, as are often seen in this process.

*RPGR* (MIM 312610) has at least 10 alternatively spliced transcripts, in which the constitute RPGR^const^ and *RPGR*^ORF15^ are two major identified RPGR isoforms. *RPGR*^const^ has 19 exons and encodes a 90-kD RPGR protein including an N-terminal domain with a tandem repeat structure similar to the regulator of chromosome condensation (RCC1) and a C-terminus with an isoprenylation site^33^, whereas *RPGR*^ORF15^ terminates in a large, purine-rich, alternative ORF 15 exon. RPGR^ORF15^ shares RCC1 domains at its N-terminus with RPGR^const^, and has a Glu-Gly rich domain encoded by exon ORF15 at its C-terminus^34^. RPGR^const^ is expressed in a broad range of ciliated tissues, whereas RPGR^ORF15^ is preferentially expressed in the connecting cilia of photoreceptor cells in the retina^35^.

RPGR^ORF15^ can interact with a series of proteins including PrBP/d, RPGRIP1, NPM1,SMC1 and SMC3, and plays an important role in ciliogenesis, photoreceptor integrity and protein trafficking in the connecting cilia of the photoreceptor cells^36^. Mutations in *RPGR*^ORF15^ may cause X-linked retinitis pigmentosa, macular dystrophy (XLCRD), cone dystrophy (XLCD) and atrophic macular degeneration^14^,^34^,^37^,^38^,^39^,^40^. Exon ORF15 is regarded as a hotspot for mutations because 50–60% mutations are clustered in this exon, where frameshift mutations are usually common due to its purine-rich structure that may promote polymerase arrest and slipped strand mispairing events^41^,^42^. Frameshift mutations may result in truncated products of different lengths of RPGR^ORF15^, which could affect RPGR^ORF15^ glutamylation at its Glu-Gly rich domain. It is suggested that the glutamylated RPGR^ORF15^ regulates the integrity of the multiprotein complexes at the cilium and modulates their entry or retention inside the cilium. The impaired RPGR^ORF15^ would decrease its stability and interactions with other proteins. The shorter the sequence of the ORF15, the lower are the binding ability with other proteins and glutamylation reaction^43^.

The indel mutations c.2002dupC (p.H668PfsX4), c.2236_2237delCT (p.E746fs22), and c.2899delG (p.F967LfsX121) are located in the glutamic-acid rich domain of RPGR^ORF15^. These mutations would produce a truncated protein due to a frameshift change of *RPGR* gene, which could be predicted to decrease their binding ability to other proteins. A previous study showed that a Glu1071X mutation in the ORF15 could decrease the reactive ability of RPGR^ORF15^ toward GT333 more than 50%, whereas RPGR^ORF15^ carrying with Glu853X mutation could keep only 0.1% binding ability to other proteins^44^. Either the p.H668PfsX4 or p.E746fs22 mutation would produce a much more truncated amino acid fragment than Glu853X did, and would be predicted to have much stronger destructive power to the RPGR^ORF15^ glutamylation and binding activity than Glu853X did. The splicing mutation c.1059 + 1 G > T in the intron 9–10 produced a variation score of −29.38 calculated by the HSF program and would be predicted to result in a donor splice site broken and a frameshift change of *RPGR* gene.

In summary, we investigated the gene mutations for five Chinese families with X-linked retinitis pigmentosa and identified three novel frameshift mutations in *RPGR* gene including c.1059 + 1 G > T, c.2002dupC (p.H668PfsX4), c.2236_2237delCT (p.E746fs22) and a previously reported mutation of c.2899delG (p.F967LfsX121). In addition, we also detected a rare large deletion in *RP2* gene. These mutations would expand the mutation spectrum of *RP2* and *RGPG*, and help to study molecular pathogenesis of RP.

### Web sites

NCBI: http://www.ncbi.nlm.nih.gov/Database/index.html; Genethon: http://www.genethon.fr/; UCSC Genome Browser: http://www.genome.ucsc.edu/; International Society for Clinical Electrophysiology of Vision: http://www.iscev.org.

## Additional Information

**How to cite this article:** Jiang, J. *et al*. Analysis of *RP2* and *RPGR* Mutations in Five X-Linked Chinese Families With Retinitis Pigmentosa. *Sci. Rep.* **7**, 44465; doi: 10.1038/srep44465 (2017).

**Publisher's note:** Springer Nature remains neutral with regard to jurisdictional claims in published maps and institutional affiliations.

## Supplementary Material

Supplementary Table 1

## Figures and Tables

**Table 1:** The primer pairs for amplification of the individual exons of *RPGR* and *RP2.*

Gene	Exon	Forward	Reverse	Size(bp)
RPGR	1	AGCGCGGTTGTAGTTGATCT	TCCTCTGCCCTCAGTCATTC	342
	2	TGTCATGAGACAAGGGGTTTG	TGTCACTGCTTTTCTGTGTTGA	587
	3	GCTTTGTGGTGACCTCATCTT	CCTTTGTGGTCCCTGGATTT	385
	4	CCCGCTATTAAATCTCGGTTC	TGCAAAGGCAAACGTGTACT	322
	5	GGCCTTGCTTGTTTTGCTT	GGAAAGGAATGTGTCCCAGA	377
	6	CAATCAGGCTGTTCTGTGTTC	CATGGACAACCATGGCATTA	482
	7	GGGATACCATAGGGAGCAAGA	TCATTAGCCACCACAGAACG	543
	8	GGTTGAACGTGACAGTTTTTCC	CATCGGCCTATTGTGAGGTT	474
	9	GATGTTACATGCAGGACCACA	GGAGGAAAATTAGGAGCAACAC	500
	10	GTGGAGTGTTGGCATACTTTGA	AGGAGCACTGATGGTGCTTT	475
	11	TCCCTGACATGAGGTTAAAGG	CCGCTCTCAATTGCCATACT	440
	12	GGCAATTGAGAGCGGATTC	GAACAAGGCAAAATGGAGGT	400
	13	AGAGAGTGGCACAAATGATCC	TGTGTCCTCCATCACTTTCCT	505
	14	CGGTATGGCAGGAAATTGAT	TCATCTTGCCAGTGTTCTGC	365
	15	GTGGCATGCGTAAAATCCTT	TTTCTGATCCCAAAGGCAAC	488
	16	GCATCTAAGGCCCCTCACTT	TGTGAAGAAGCTGTGGTGGT	435
	17	ACTCCAGGGGTGAGCTCTTT	TTTGGGGCCATGAAAACTC	331
	18	ATGTGGTGCCCCATAAAGAG	TGAACAGTTAAGGCCACAACAC	574
	ORF15-1	AGGAAGGAGCAGAGGATTCA	CCCTCTTCTTCCATTCTTCC	348
	ORF15-2	GGGGAGAAAGACAAGGGTAG	TCCTTTCCCCTCCTCTACTT	444
	ORF15-3	GGAAGAAGGAGACCAAGGAG	CCCATTTCCCTGTGTGTTAG	982
	ORF15-4	GCAGGATGGAGAGGAGTACA	GAGAGAGGCCAAAATTTACCA	415
RP2	1	AAAAAGGGGAAGGGGTGCT	AGCTATCCGCGTTCAAGAGA	397
	2A	CTGGCAGCCAATAGTCCTTT	TCTTCTGGAAGAAGGCTCCA	551
	2B	TTTCCGGAATTGCAGAGATT	TGAGGGTTGCCTGTATTTCC	575
	3	TCAGTGTGCTGTTGTTGCAT	AGGGCTCCTTGAGTGATGTG	393
	4	CCCTCAAAGAAGATGCTGGA	TCCAAGCAACATTAGGGACA	294
	5	CTGTGGAGAACATGGGCTTT	TCATGGAGCACTGAGGAAAGT	521
