# Supplementary material for: Analysis of RP2 and RPGR Mutations in Five X-Linked Chinese Families with Retinitis Pigmentosa
Source: Sci Rep. 2017 Mar 15;7:44465. doi: 10.1038/srep44465 (PMC5353642; doi:10.1038/srep44465)
Supplement: Supplementary Table 1 [file srep44465-s1.doc]

Table 1. Clinical Characteristics of all affected members in all five XLRP families

| **Family**  **idification** | **Individual**  **number** | **Age/Sex** | **Onset (years)** | **Visual Acuity** | **Fundus** | **Total mERG Amplitude (nv/deg)** |
| --- | --- | --- | --- | --- | --- | --- |
| XLRP001 | Ⅰ:2 | 67/F | NA | OD 0.1 | OD BP,AN,OA | NA |
|  |  |  |  | OS 0.2 | OS BP,AN,OA | NA |
|  | Ⅱ:3 | 38/M | 7-8 | OD 0.01 | OD BP,AN,OA | EXTINGUISHED |
|  |  |  |  | OS 0.02 | OS BP,AN,OA | EXTINGUISHED |
|  | Ⅱ:5 | 32/F | NA | OD 0.7 | OD BP,AN | DECREASED |
|  |  |  |  | OS 0.8 | OS BP,AN | DECREASED |
|  | Ⅱ:6 | 30/M | 7 | OD 0.04 | OD BP,AN,OA | EXTINGUISHED |
|  |  |  |  | OS 0.02 | OS BP,AN,OA | EXTINGUISHED |
|  | Ⅲ:1 | 10/M | 8 | OD 0.3 | OD BP, AN | DECREASED |
|  |  |  |  | OS 0.2 | OS BP, AN | DECREASED |
| XLRP002 | Ⅰ:2 | 76/F | NA | OD 1.0 | OD BP,AN,T | DECREASED |
|  |  |  |  | OS 1.0 | OS BP,AN,T | DECREASED |
|  | Ⅱ:2 | 40/F | NA | OD 0.7 | OD BP, AN，T | NA |
|  |  |  |  | OS 0.4 | OS BP, AN，T | NA |
|  | Ⅱ:6 | 36/F | NA | OD 0.5 | OD BP, AN，T | DECREASED |
|  |  |  |  | OS 0.3 | OS BP, AN，T | DECREASED |
|  | Ⅲ:1 | 10/M | 5 | OD 0.3 | OD BP, AN | DECREASED |
|  |  |  |  | OS 0.3 | OS BP, AN | DECREASED |
|  | Ⅲ:2 | 6/M | 4 | OD 0.4 | OD BP, AN | NA |
|  |  |  |  | OS 0.4 | OD BP, AN | NA |
|  | Ⅲ:5 | 7/M | 4 | OD 0.2 | OD BP, AN | DECREASED |
|  |  |  |  | 0S 0.3 | OD BP, AN | DECREASED |
| XLRP003 | Ⅰ:1 | 65/F | NA | OD 0.4 | OD BP, AN，T | NA |
|  |  |  |  | OS 0.2 | OS BP, AN，T | NA |
|  | Ⅱ:1 | 41/M | 7-8 | OD FC | OD BP,AN,OA | EXTINGUISHED |
|  |  |  |  | OS .FC | OS BP,AN,OA | EXTINGUISHED |
|  | Ⅲ:3 | 10/F | NA | OD 1.0 | OD BP | NA |
|  |  |  |  | OS 1.0 | OS BP | NA |
| XLRP004 | Ⅰ:1 | 57/M | 7-8 | OD HM | OD BP,AN,OA | EXTINGUISHED |
|  |  |  |  | OS LP | OS BP,AN,OA | EXTINGUISHED |
|  | Ⅱ:1 | 33/F | NA | OD 0.7 | OD BP,AN | DECREASED |
|  |  |  |  | OS 0.8 | OS BP,AN | DECREASED |
|  | Ⅲ:1 | 8/M | 6 | OD:0.2 | OD BP, AN | DECREASED |
|  |  |  |  | OS:0.3 | OS BP, AN | DECREASED |
| XLRP005 | Ⅱ:1 | 35/M | 7-8 | OD 0.01 | OD BP,AN,OA | EXTINGUISHED |
|  |  |  |  | OS 0.05 | OS BP,AN,OA | EXTINGUISHED |
|  | Ⅲ:3 | 6/M | 5 | OD 0.4 | OD BP, AN | DECREASED |
|  |  |  |  | OS 0.4 | OS BP, AN | DECREASED |

FC, finger count

HM, hand move

LP, light perception

BP, bone spicule-like pigment deposit

OA, optic atrophy ，

AN, narrowing of the retinal vessel

T, tapetal-like reflex

NA, not available
